# Protective Effect of Joa-Gui Em through the Improvement of the NLRP3 and TLR4/NF-*κ*b Signaling by Ischemia/Reperfusion-Induced Acute Renal Failure Rats

**DOI:** 10.1155/2021/7178868

**Published:** 2021-05-28

**Authors:** Se Won Na, Youn Jae Jang, Mi Hyeon Hong, Jung Joo Yoon, Ho Sub Lee, Hye Yoom Kim, Dae Gill Kang

**Affiliations:** ^1^College of Oriental Medicine and Professional Graduate School of Oriental Medicine, Wonkwang University, Iksan 54538, Republic of Korea; ^2^Hanbang Cardio-Renal Research Center and Professional Graduate School of Oriental Medicine, Wonkwang University, Iksan 54538, Republic of Korea

## Abstract

Joa-gui em (左歸飮, JGE) is known to be effective for treating kidney-yin deficient syndrome. However, there is a lack of objective pharmacological research on improving kidney function. This study was designed to evaluate whether JGE improves renal function and related mechanisms in rats with acute renal injury induced by ischemia/reperfusion (I/R). The acute renal failure (ARF) group was subjected to reperfusion after inserting a clip into the renal artery for 45 min. The ARF + JGE (100 or 200 mg/kg/day) groups were orally administered for four days after their I/R surgery, respectively. JGE treatment suppressed the increase in kidney size in the ARF animal model and alleviated the polyuria symptoms. In addition, to confirm the effect of improving the kidney function of JGE, lactate dehydrogenase levels, blood urea nitrogen/creatinine ratio, and creatinine clearance were measured. As a result, it decreased in the ARF group but significantly improved in the JGE group. Also, as a result of examining the morphological aspects of renal tissue, it was shown that JGE improved renal fibrosis caused by ARF. Meanwhile, it was confirmed that JGE reduced inflammation through the nucleotide-binding oligomerization domain-like receptor pyrin domain containing-3 (NLRP3) and toll-like receptor 4 (TLR4)/nuclear factor kappa B (NF-*κ*B) signaling pathways, which are the major causes of acute ischemic kidney injury, thereby improving renal function disorder. The JGE has a protective effect by improving the NLRP3 and TLR4/NF-*κ*B signaling pathway in rats with acute renal dysfunction induced by I/R injury.

## 1. Introduction

The kidney is essential in maintaining homeostasis by regulating fluid volume through the excretion and reabsorption of water. Ischemic kidney injury due to ischemia/reperfusion (I/R) causes various kidney dysfunction, eventually leading to acute renal failure (ARF). Moreover, kidney function is impaired, urine cannot be excreted normally, and the body loses its balance of water and electrolytes. The rapid increase in the levels of serum creatinine (Cr) decreases glomerular filtration in the kidneys [1]. It is widely assumed that ARF generally occurs due to acute tubular necrosis, usually due to ischemic renal injury [2]. ARF animal model has structural remodeling in the renal tubular epithelium [3, 4]. I/R-induced renal impairment significantly affects kidney function because the supply of reperfusion to the kidney causes a significant cellular metabolic disturbance and tissue inflammation [5, 6]. In inflammatory kidney diseases, the nucleotide-binding oligomerization domain-like receptor pyrin domain containing-3 (NLRP3) inflammasome is a multi-protein complex induced by harmful factors in the body and plays an essential role in the inflammatory response [7, 8]. Activation of NLRP3 inflammasomes mediates the activation of caspase-1 and the secretion of inflammatory cytokines, including interleukin- (IL-) 1*β* and IL-18, resulting in a type of cell death called pyroptosis [9–11]. Therefore, the NLRP3 inflammasome and proinflammatory cytokines, including IL-1*ß* and IL-18, directly affect the renal tubular epithelium and cause renal dysfunction [12].

Some studies have reported that the NLRP3 inflammasome promotes epithelial-mesenchymal transition (EMT) by the transforming the growth factor-*β*_1_ (TGF-*β*_1_)/Smad signaling [13]. The NLRR3 inflammasome increases TGF-*β*_1_ expression, and TGF-*ß*_1_ is associated with EMT and leads to renal fibrosis [14]. EMT of renal tubular cells is defined as the process that contributes to fibrosis through a phenotypic change to myofibroblasts [15]. Furthermore, TGF-*β*_1_ mediates the formation of extracellular matrix proteins and pro-fibrotic factors, including fibronectin, collagen, and matrix metalloproteinases [16]. Like this, the relationship between NLRP3 inflammasomes and kidney disease is important, and it is necessary to confirm the efficacy of drugs to improve the related signaling pathways. Therefore, we tried to examine the efficacy of herbal medicines that have been used in traditional Korean medicine to improve kidney function.

In Korean traditional medicine, there are four categories of renal disease symptoms, including deficiency of kidney yang (腎陽虛), deficiency of kidney-yin (腎陰虛), insufficiency of kidney essence (腎精虛), and insufficiency of kidney gi (腎氣虛). Joa-gui em (左歸飮, JGE) is known to be effective in treating kidney-yin deficient syndrome [17, 18]. However, there is a lack of objective pharmacological research on improving acute renal failure (ARF) model. Therefore, this study was conducted to confirm the effect of JGE on the improvement of renal function and related mechanisms in an I/R-induced ARF animal model.

## 2. Materials and Methods

### 2.1. Preparation of Joa-Gui Em

The voucher specimen used in this study (HBI 192–15) was stored in the Hanbang Cardio-Renal Syndrome Research Center of Wonkwang University (Iksan, Korea). The herbal medicines used to extract the JGE were purchased from the Herbal Medicine Cooperative Association (Iksan, Korea). The six herbal medicines that make up JGE are as follows: Rehmannia glutinosa Libosch (80 g), Dioscorea batatas Decne (80 g), Cornus officinalis Siebold (80 g), Lycium chinense Mill (80 g), Poria cocos Wolf (60 g), and Glycyrrhiza uralensis Fisch (40 g). The herbal medicines were soaked in 2 L of distilled water and left at room temperature for 1 hour and then boiled for 2 hours (at 100°C). The boiled herbal decoction was centrifuged for 10 minutes (at 4°C, 3000 rpm) to remove impurities and concentrated in a rotary vacuum evaporator (N-11, Rikakikai, Tokyo, Japan), and then a freeze dryer was used to create a powder. Dried JGE was stored at 4°C until use, and for in vivo experiments, it was diluted in distilled water at an appropriate dose before oral administration and used.

### 2.2. Animals

Sprague-Dawley male rats (5 weeks old, weight 170–190 g) were purchased from Samtako (Samtako Bio Korea, Osan, Korea) and maintained in a 12 hr light/dark cycle in a thermo-hygrostat (45% humidity). To make an animal model of ARF induced by I/R, anesthesia was performed using sodium pentobarbital (50 mg/kg, intraperitoneal injection) and surgery was performed. Anesthetized rats induced blocking both renal arteries with clips to prevent blood from passing through them for 45 minutes, and control rats underwent sham surgery without clips. Animals recovered in metabolic cage for 4 days. It was divided into 4 groups as follows: Cont, control group; ARF, ARF group; ARF + JGE100, JGE 100 mg/kg/day-treated ARF group; ARF + JGE 200, JGE 200 mg/kg/day-treated ARF group. Rats were administered JGE by oral gavage for 7 days. This study was tested on animals with the approval of the Institutional Animal Care and Use Committee (IACUC) of Wonkwang University (WKU19-46).

### 2.3. Renal Function Test

Rats in each group were measured for water intake and quantitative urine collection in separate metabolic cages (24 hr). Urine osmolality (Model 3900, Advanced Instruments Inc., Norwood, MS, USA), and electrolytes (NOVA 4, Biochemical, Waltham, MA, USA) levels were measured using the collected urine. After the experiment was completed, the blood of the experimental animals was taken to measure lactate dehydrogenase (LDH), blood urea nitrogen (BUN), and Cr in the plasma. After the experiment was completed, the experimental animal was blood was taken, and the BUN, Cr, and LDH in the plasma were measured using biochemical analyzer (NX700, FUJIFILM Corporation, Tokyo, Japan). Plasma and urine creatinine clearance (Ccr) were measured with a spectrophotometer using a colorimetric method (Milton Roy, Rochester, NY, USA). Ccr (ml/min/kg) = urine Cr (mg/ml) *x* urinary volume (UV, ml/kg/min)/plasma Cr (mg/ml).

### 2.4. Western Blot Analysis

Protein samples (30 *μ*g protein) were electrophoresed and transferred to a nitrocellulose membrane. The membrane was then blocked in 5% bovine serum albumin (with Tris-buffered saline) for 2 hours and then incubated with an appropriate primary antibody. The next day, the secondary antibody was reacted for 1 hour and then visualized using chemiluminescence (EzWestLumi plus, ATTO Technology, NY, USA). Primary antibodies included cryopyrin NLRP3, apoptosis-associated speck-like protein containing a caspase recruitment domain (ASC), pro-caspase-1, TGF-*ß*_1_, IL-1*β*, toll-like receptor 4 (TLR4), myeloid differentiation primary response gene 88 (MyD88), nuclear factor kappa B (NF-*κ*B) p65, and *β*-actin (Santa Cruz Biotechnology Dallas, TX, USA). Protein expression levels were imaged using iBright FL100 image analyzer (Thermo Fisher Scientific, Waltham, MA, USA).

### 2.5. Histopathologic Examination

Kidney tissues were isolated from each rat and fixed with 10% paraformaldehyde in phosphate-buffered saline (PBS, 0.01 M) for 24 hr. The kidney tissues were dehydrated with a sequence of ethanol solutions and embedded in paraffin (sectioned on slides, 6 *μ*m). For histopathological comparison, sectioned kidneys were stained with hematoxylin and eosin (H&E), periodic acid shift (PAS), picrosirius red, and Masson's trichrome staining imaged using a EVOS^TM^ light microscope (M5000, Thermo Fisher Scientific, Bothell, WA, USA).

### 2.6. Statistical Analyses

All results were presented as mean ± SEM. Statistical significance between groups was performed using ± standard error (SE). *p* value < 0.05 was considered statistically significant. The significant differences between groups were validated by paired *t*-test. All statistical analyses were conducted using SigmaPlot 10.0.

## 3. Results

### 3.1. High-Performance Liquid Chromatography (HPLC) Analysis of JGE

**Figure 1:**
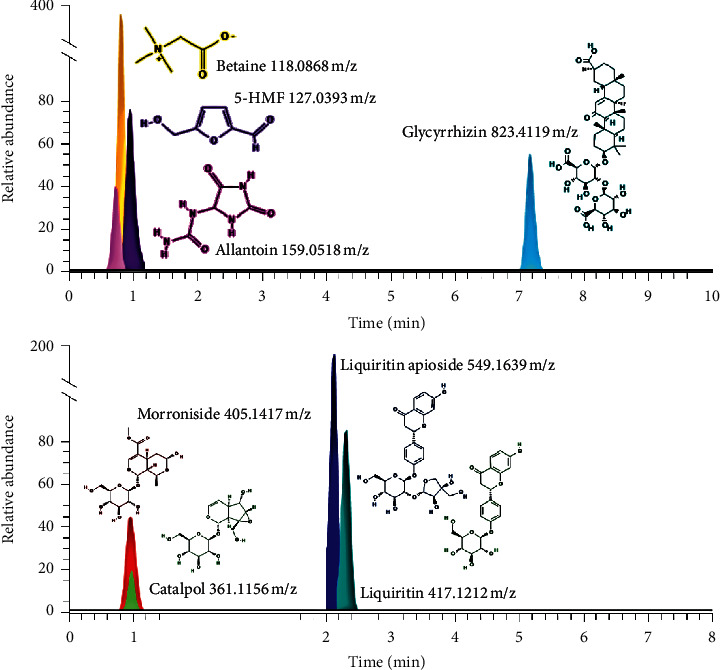
Three-dimensional chromatogram of JGE obtained using HPLC-PDA. Extracted ion chromatograms from the ultra-performance liquid chromatography analysis of charged molecular ions, showing eight bioactive compounds.

The chemical composition of JGE was analyzed using ultra-high performance liquid chromatography. Fifteen compounds, including eight markers (5-hydroxymethyl furfural [5-HMF], allantoin, betaine, catalpol, cocamidopropyl betaine, glucose, glycyrrhizin, liquiritin, liquirtin apioside, maltotriose, morroniside, phenylalanine, quinic acid, sucrose, tryptophan, and valine) were verified based on the authentic compounds or tentatively identified according to the retention time, exact mass spectrometry (MS), and MS/MS fragments. In particular, major bioactive compounds such as 5-HMF and catalpol for *Rehmanniae Radix Preparata*, allantoin for *Dioscoreae Rhizoma*, betaine for *Lycii Fructus*, morroniside for *Corni Fructus*, glycyrrhizin, liquiritin, and liquiritin apioside for *Glycyrrhizae Radix et Rhizoma* were detected in JGE (Figure 1).

### 3.2. Effect of JGE on Physical Measurements

I/R injured rats are commonly used as a model for experimental ARF research. The body weights (BWs) of all rats in the ARF group were significantly decreased and were markedly restored by oral administration of JGE200 (*p* < 0.05) (Table 1). The kidney weight/BWs of the I/R injury rats treated with JGE were significantly decreased compared with the non-JGE treated I/R injury rats (Table 1). However, there was no difference between the ARF and JGE-treated groups in terms of heart weight/BW (Table 1).

### 3.3. Effect of JGE on Urinalysis for Kidney Function

ARF is characterized by a sudden loss of kidney function in concentrating urine. In order to investigate the change in urine and water intake of JGE, the rats of each group were kept in separate metabolic cages for 4 days, and urine samples were collected. UV was significantly increased in the ARF group, which was markedly decreased by oral administration of JGE100 (*p* < 0.05) and JGE200 (*p* < 0.01) on day 4 (Table 2). These results indicated that the ability to concentrate urine was impaired due to the I/R injury. However, JGE treatment did not significantly change urinary osmolality in ARF rats (Table 2). Urinary sodium excretion was decreased by the I/R injury and was markedly restored by the oral administration of JGE200 (*p* < 0.05) on day 4 (Table 2). Urinary potassium excretion was reduced in ARF rats, which was restored significantly by oral administration of JGE100 (*p* < 0.05). Urinary chloride excretion was decreased in ARF rats, which was markedly restored by oral administration of JGE100 (*p* < 0.05).

### 3.4. Effect of JGE on Renal Functional Parameters

BUN/Cr ratio was significantly increased in ARF rats compared with control rats. BUN/Cr was significantly decreased by the oral administration of JGE in a dose-dependent manner (Table 3). Ccr was significantly lower in the ARF group than in the control group. Oral administration of JGE significantly restored Ccr (Table 3). In addition, we measured the effect of JGE on the plasma levels of LDH in ARF rats. As a result, LDH levels were increased by I/R injury and significantly decreased by the oral administration of JGE100 (*p* < 0.01) and JGE200 (*p* < 0.001) (Table 3).

### 3.5. Effect of JGE on Histological Changes in Kidney

**Figure 2:**
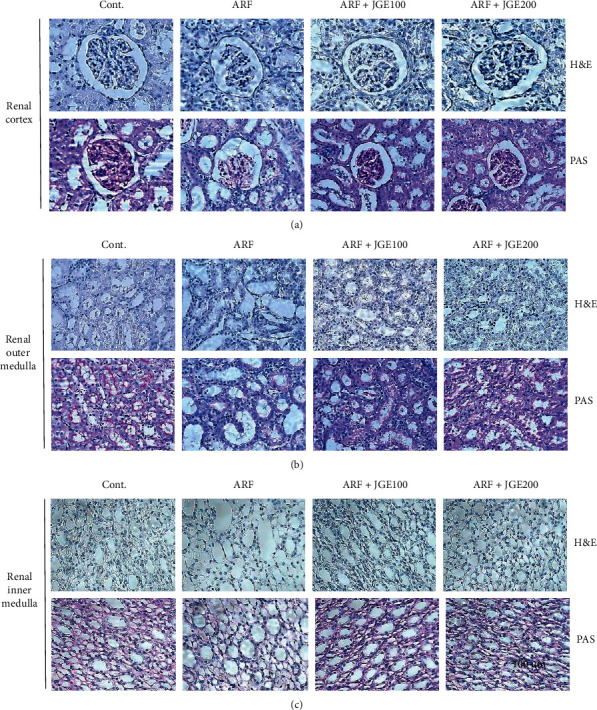
Effect of JGE on the renal cortex (a), outer medulla (b), and inner medulla (c). Sections of the kidney are demonstrated from the control, I/R-induced ARF, and JGE (100 or 200 mg/kg/day)-treated ARF group. Representative microscopic photographs were stained using H&E and PAS (magnification 400 ×).

To determine the protective effect of JGE on renal cortical glomeruli injury, renal morphology was analyzed using H&E and PAS staining. Light microscopic examinations showed glomerular injury and vacuole formation in the renal cortex of rats in the ARF group. However, JGE treatment improved the glomerular injury of the renal cortex (Figure 2(a)). H&E and PAS staining were performed to confirm the protective effect of JGE on the extrarenal medulla and inner medulla damage, and histological analysis was performed, and representative micrographs of the kidneys of each group were obtained. It has been demonstrated that tubular dilatation, tubular epithelial damage, cast formation, and debris accumulation were found in the external and internal water quality of ARF (Figure 2(b)). The renal outer medulla and inner medulla tubules destroyed by I/R injury were treated with JGE to prevent the lesions of the tubules (Figure 2(c)). These results imply that JGE has the effect of improving pathological damage to the kidney in I/R-induced ARF rats.

### 3.6. Effect of JGE on NLRP3 Inflammasome Expression in Kidney

**Figure 3:**
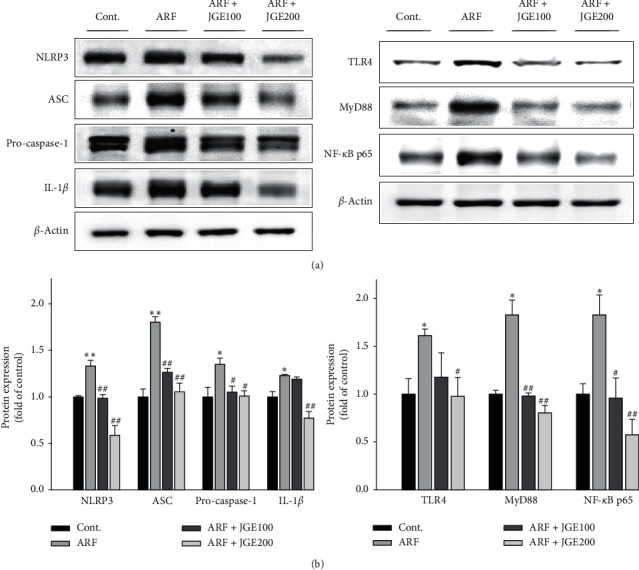
Effect of JGE on proinflammatory cytokine and NLRP3 inflammasome in kidney tissues. Protein expression of NLRP3 inflammasomes, including NLRP3, pro-caspase-1, and ASC (a), and proinflammatory cytokine IL-1*β*, and the TLR4/MyD88/NF-*κ*B signaling pathway (b) in kidneys were analyzed by western blot analysis. The data shown summarize three independent experiments. Values are expressed as means ± SE.  ^*∗*^*p* < 0.05,  ^*∗∗*^*p* < 0.01 vs. control;  ^#^*p* < 0.05,  ^##^*p* < 0.01 vs. ARF.

The NLRP3 inflammasome signaling pathway plays an essential role in renal inflammation. To evaluate the effect of JGE on the NLRP3 inflammasome induced by I/R injury, NLRP3 inflammasome protein expression was decided by western blot analysis. NLRP3 inflammasome signaling factors, NLRP3, IL-1*β*, pro-caspase-1, and ASC protein expression, increased in response to I/R-induced ARF rats, but decreased by JGE treatment (Figure 3(a). In addition, the NLRP3 inflammasome binds to the NF-*κ*B inflammatory pathway to mediate IL-1*β* transcription and activation. JGE treatment decreased the protein expression associated with the TLR4/MyD88/NF-*κ*B inflammatory pathway (Figure 3(b)).

### 3.7. Effect of JGE on Renal Fibrosis

**Figure 4:**
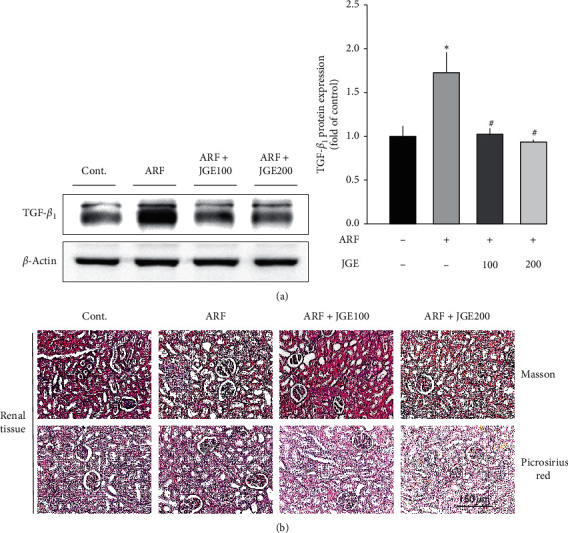
Effect of JGE on renal fibrosis. Protein expression of TGF-ß1 in the kidney tissues was determined by western blot analysis (a). The data shown summarize three independent experiments. Representative microscopic photographs were stained using Masson and PAS (b) (magnification 200 ×). Values are expressed as means ± SE.  ^*∗*^*p* < 0.05 vs. control;  ^#^*p* < 0.05 vs. ARF.

To confirm the protective effect of JGE on renal fibrosis, histological analysis was performed by staining with Masson Trichrome and picrosirius red. In addition, TGF-*ß*_1_ is a major profibrotic cytokine that drives EMT. To investigate the effect of JGE on renal fibrosis induced by I/R injury, TGF-*ß*_1_ protein expression was determined (Figure 4(a)). It was known that TGF-*ß*_1_ expression is increased in response to NLRP3 inflammasome. As shown in Figure 4(a), I/R enhanced TGF-*ß*_1_ protein expression, whereas it was inhibited by JGE treatment. In addition to determine the protective effect of JGE on renal fibrosis, renal morphology was analyzed using Masson and PAS staining. The renal fibrosis destroyed by I/R injury was treated with JGE to prevent the lesions of the tubules (Figure 4(b)). Thus, JGE suppressed I/R-induced renal fibrosis in ARF rats.

## 4. Discussion

JGE was recorded in a traditional Chinese medical book titled “Gyeong-agjeonseo” and comprises six components of herbal medicines*: Rehmanniae Radix Preparat, Dioscoreae Rhizoma, Lycii Fructus, Hoelen, Corni Fructus*, and *Glycyrrhizae Radix Praeparata.* JGE has been used for the treatment of kidney-yin-deficient syndrome [17, 18]. However, there is no evidence that JGE has an effect on I/R-induced ARF. Therefore, this study demonstrated whether JGE improves renal dysfunction of rats with I/R-induced ARF.

In this study, urine volume increased in renal function in I/R-induced ARF rats, while Ccr and excretion of sodium, potassium, and chloride were significantly decreased. These results indicate that ARF induced by I/R caused a defect in the rat with the ability to concentrate urine [19]. ARF is one of the most common glomerular diseases characterized by a marked decrease in glomerular filtration rate, extensive tubular cell necrosis, glomerular damage, and signs of tubular obstruction due to cell debris [20–22]. It is well documented that ARF is characterized by an acute decline in renal function as measured by UV, Ccr, and excretion of sodium, potassium, and chloride [23–25]. In this study, JGE treatment ameliorated renal function, such as Ccr, UV, and the excretion of sodium, potassium, and chloride in I/R-induced ARF rats. These findings suggest that JGE has a potential role in renal dysfunction.

It has been established that tubular dilatation, tubular epithelial damage, cast formation, and debris accumulation were all found in the renal medulla of ARF [9, 26, 27]. To confirm the effect of JGE administration on tubular injury, PAS staining analysis was performed. The present study showed that the cortex, inner medulla, and outer medulla were impaired in rats with ischemic acute kidney injury. In the present study, JGE treatment ameliorated renal injury, including tubular dilatation, tubular epithelial damage, cast formation, and debris accumulation in I/R-induced ARF rats. Furthermore, glomerular injury was also found, and vacuole formation was observed in the cortices of the kidneys. The present data showed that JGE ameliorated glomerular injury by inhibiting vacuole formation in rats with ischemic acute kidney injury.

In a previous study, the elevation of BUN/Cr and levels of LDH in serum was pathognomonic for I/R-induced ARF. This study showed the same results of earlier studies, which reported that the parameters of renal function, including the levels of BUN, Cr, and LDH, are decreased by I/R injury [28]. This study aimed to determine if JGE treatment inhibited renal failure. Therefore, the present study shows that JGE treatment ameliorates renal derangement, as observed by the changes in BUN/Cr and levels of LDH in I/R-induced ARF rats.

**Figure 5:**
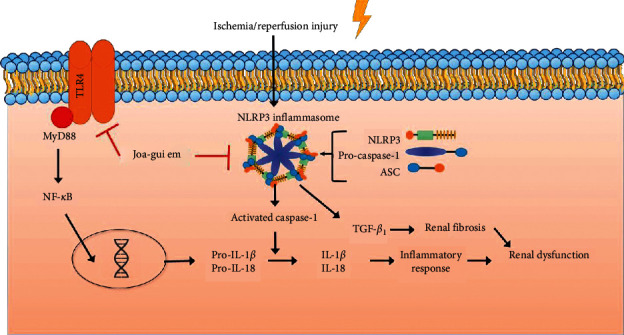
The suggested mechanism of the anti-inflammatory and anti-fibrotic effects of JGE in I/R-induced ARF rats.

In another study of I/R-induced ARF, it was concluded that acute kidney injury causes the NLRP3 inflammasome to be released by directly affecting the renal tubular epithelium, which leads to the activation of caspase-1, causing inflammatory cell infiltration and the activation of cytokines [29–31]. Activated caspase-1 of the NLRP3 inflammasome activates IL-1*β* and IL-18 [32–34]. In our present study, as a result of Western blot analysis, it was revealed that JGE reduced NLRP3 inflammasome formation in I/R-induced ARF rats. Also, we showed that JGE significantly inhibited the NLRP3 inflammasome in I/R-induced ARF rats of pro-inflammatory cytokines such as NLRP3, ASC, pro-caspase-1, and IL-1*β*. Therefore, these results suggest that JGE reduces NLRP3 inflammasome formation, thereby reducing the inflammatory response. Furthermore, the NLRP3 inflammasome activates proinflammatory cytokines that are associated with the TLR4/MyD88/NF-*κ*B signaling. Widely expressed in the plasma membrane of immune cells, TLR4 plays a vital role in initiating inflammation, and MyD88 is used by all TLRs and activates NF-*κ*B for induction of inflammatory cytokine genes [35, 36]. The present results showed that JGE treatment significantly suppressed the protein expression of TLR4, MyD88, and NF-*κ*B in I/R-induced ARF rats. Therefore, we speculated that JGE exhibits anti-inflammatory effects by inhibiting the NLRP3 inflammasome and TLR4/MyD88/NF-*κ*B signaling pathway (Figure 5).

Some studies have reported that NLRP3 inflammasomes are required for optimal TGF-*ß*_1_ signaling and R-Smad activation [37]. In the absence of NLRP3, TGF-*ß*_1_ signaling is interrupted in the renal tubular epithelium, and the expression of TGF-*ß*_1_-stimulated genes, which are important for EMT, is reduced [38]. EMT of renal tubular cells is defined as obtaining myofibroblast markers, producing extracellular matrix proteins, and migratory capabilities instead of losing epithelial characteristics, such as tight junction formation and apical-basal polarity [39]. TGF-*ß*_1_ is the major profibrotic cytokine that drives the course of EMT [40]. In this study, JGE treatment reduced the protein expression of TGF-*β*_1_ in I/R-induced ARF rats. These results suggest that JGE has a potential role in renal fibrosis.

## 5. Conclusion

In conclusion, treatment with JGE has protective effects against renal dysfunction induced by I/R injury via inhibition of the NLRP3 signaling pathway, indicating that JGE should be considered an effective Korean traditional medicine for treating acute kidney injury and renal remodeling.

## Figures and Tables

**Table 1:** Effect of JGE on physical measurements.

	Cont	ARF	ARF + JGE100	ARF + JGE200
BW (g)	217.90 ± 2.07	191.63 ± 2.60^∗∗∗^ ^*∗∗∗*^	197.27 ± 2.44	202.24 ± 3.13 ^#^
KW/BW (g/kg)	8.37 ± 0.04	12.53 ± 0.35^∗∗∗^ ^*∗∗∗*^	11.46 ± 0.30 ^#^	10.39 ± 0.53 ^##^
HW/BW (g/kg)	3.66 ± 0.02	3.49 ± 0.03^∗∗∗^ ^*∗∗∗*^	3.55 ± 0.03	3.56 ± 0.04

Cont., control; ARF, ischemia/reperfusion- (I/R-) induced acute renal failure (ARF) group; ARF + JGE100, JGE 100 mg/kg/day; ARF + JGE200, JGE 200 mg/kg/day for 4 days; KW: kidney weight; HW: heart weight; BW: body weight. Values are expressed as mean ± SE. (*n* = 10).^∗∗∗^ ^*∗∗∗*^*p* < 0.001 vs. cont.;  ^#^*p* < 0.05,  ^##^*p* < 0.01 vs. ARF.

**Table 2:** Effect of JGE on urinalysis for kidney function.

	Cont	ARF	ARF + JGE100	ARF + JGE200
UV (ml/day/kg)	45.41 ± 2.06	70.66 ± 3.28^∗∗∗^ ^*∗∗∗*^	61.31 ± 2.71 ^#^	57.92 ± 1.77 ^##^
Uosmol (mOsm)	2503.8 ± 131.8	1420.5 ± 86.8^∗∗∗^ ^*∗∗∗*^	1643.1 ± 114.8	1615.8 ± 62.3
U_Na_V (mmol/L/Kg)	786.75 ± 19.82	485.24 ± 30.09^∗∗∗^ ^*∗∗∗*^	566.01 ± 30.92	606.73 ± 39.40 ^#^
U_K_V (mmol/L/Kg)	1556.98 ± 51.95	995.33 ± 64.84^∗∗∗^ ^*∗∗∗*^	1302.99 ± 99.76 ^#^	1101.53 ± 66.83
U_Cl_V (mmol/L/Kg)	1324.03 ± 37.16	871.52 ± 46.16^∗∗∗^ ^*∗∗∗*^	1044.75 ± 64.87 ^#^	968.47 ± 61.45

Cont, control; ARF, ischemia/reperfusion- (I/R-) induced acute renal failure (ARF) group; ARF + JGE100, JGE 100 mg/kg/day; ARF + JGE200, JGE 200 mg/kg/day for 4 days; UV, urinary volume; Uosmol, urinary osmolality. Values are expressed as mean ± SE. (*n* = 10). cont^∗∗∗^ ^*∗∗∗*^*p* < 0.001 vs. cont.;  ^#^*p* < 0.05,  ^##^*p* < 0.01 vs. ARF.

**Table 3:** Effect of JGE on renal functional parameters.

	Cont	ARF	ARF + JGE100	ARF + JGE200
BUN/Cr	31.86 ± 1.14	52.27 ± 1.73^∗∗∗^ ^*∗∗∗*^	43.82 ± 2.13 ^##^	43.46 ± 0.88 ^###^
Ccr (ml/min/kg)	0.46 ± 0.03	0.26 ± 0.02^∗∗∗^ ^*∗∗∗*^	0.33 ± 0.03 ^#^	0.34 ± 0.02 ^##^
LDH (U/L)	1612.7 ± 150.7	2089.2 ± 136.6^∗^ ^*∗*^	1474.5 ± 108.6 ^##^	1104.9 ± 102.7 ^###^

Cont., control; ARF, ischemia/reperfusion- (I/R-) induced acute renal failure (ARF) group; ARF + JGE100, JGE 100 mg/kg/day; ARF + JGE200, JGE 200 mg/kg/day for 4 days; Cr, creatinine; Ccr, creatinine clearance; BUN, blood urea nitrogen; LDH, lactate dehydrogenase. Values are expressed as mean ± SE. (*n* = 10).  ^*∗*^*p* < 0.05^∗∗∗^ ^*∗∗∗*^*p* < 0.001 vs. cont.;  ^#^*p* < 0.05,  ^##^*p* < 0.01,  ^###^*p* < 0.001 vs. ARF.

## Data Availability

The datasets used and/or analyzed during the current study are available from the corresponding author on reasonable request.

## Abbreviation

ARF: Acute renal failure
BUN: Blood urea nitrogen
Cr: Creatinine
Ccr: Creatinine clearance
H&E: Hematoxylin and eosin
IL-1*β*: Interleukin-1*β*
I/R: Ischemia/reperfusion
JGE: Joa-gui em
LDH: Lactate dehydrogenase
MyD88: Myeloid differentiation primary response gene 88
NF-*κ*B: Nuclear factor kappa B
NLRP3: Nucleotide-binding oligomerization domain-like receptor pyrin domain containing-3
PAS: Periodic acid shift
TGF-*β*1: Transforming the growth factor-*β*1
TLR4: Toll-like receptor 4
